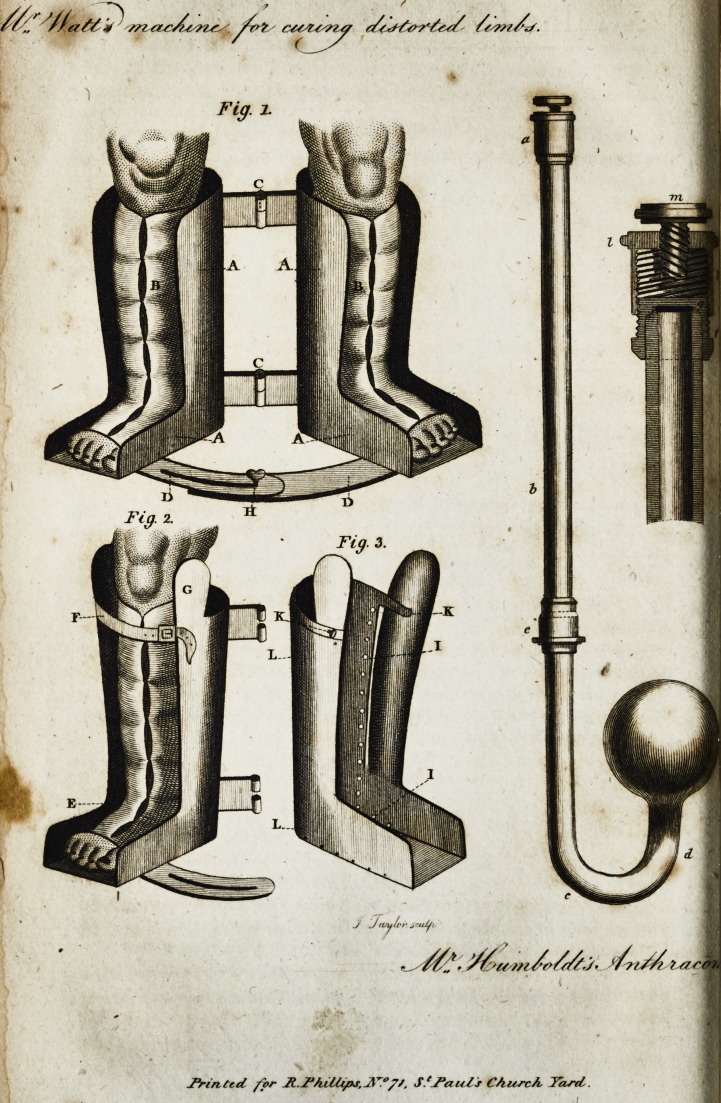# Description of a New Machine for Curing Distorted Limbs

**Published:** 1800-08

**Authors:** Robert Watt

**Affiliations:** Surgeon, in Paisley


*Mr. Watt's machine for curing distorted limbs.*

Fig. 1.

J Taylor sculp.

*Mr. Humboldt's Anthracom*

*Printed for R. Phillips, No. 71, St. Paul's Church Yard.*

THE

### Medical and Phyfical Journal.

VOL. IV.] AUGUST, 1800. [NO. XVIII.

### *Description of a new Machine for curing distorted Limbs.*

[ With an Engraving. ]

Communicated by Mr. ROBERT WATT, Surgeon, in PAISLEY.

*To the Editors of the Medical and Phyfical Journal.*

GENTLEMEN,

IN the Preface to the firft volume of your Medical and Phyfical Journal, you mention your not being fo fortunate in the departments of Anatomy and Surgery, as in the other branches of medical fcience. This may be owing to thefe two having arrived nearer to perfection than any of the reft; or, it may be owing to the negligence of fome practitioners, in not communicating freely to the world whatever chance may have thrown into their way. Others there are, who, actuated by a more felfifh and degrading principle, wifh carefully to conceal from the public, as far as poffible, any improvement or difcovery they may have made, in order that none but themfelves may reap the benefit. The idea, however, is abfurd, and equally injurious to the perfon himfelf, as to the public; for it frequently happens, that the more freely a man communicates his improvements and difcoveries to the world, the more generoufly will he be rewarded. At any rate, fuch perfons feel a reward in their breaft, of which none but themfelves can have any conception. It fhall, therefore, be my ambition, through the excellent medium of your Journal, to communicate to the public whatever hints or improvements I may in future be able to make.

There is one malady to which the human race is not infrequently fubjected, and for which, fo far as I know, furgery has made little or no provifion, namely, the diftorted limbs of infants. What I chiefly refer to, are thofe two fpecies of diſtortion, known by the terms varus and valgus. Whether the fault has been owing to the parents of theſe unfortunate children, in not having applied in time; or, whether it has been owing to a deficiency in the medical aſſiſtance they have employed, it is, perhaps, impoſſible to determine; but certain it is, that numbers of both ſexes have remained in this unhappy ſituation for life: whereas, in this part of the country, I have not ſeen a ſingle inſtance of one who has not been cured of the diſeaſe.

It is more than probable, that in many of theſe caſes the fault has been owing to the parents. They, as if they looked upon themſelves as the cauſe of the misfortune, and of courſe blameable, carefully conceal the matter as long as poſſible, not only from the public, but frequently even from their family practitioner. It is alſo a commonly received opinion, that the regular bred ſurgeon can afford little or no aſſiſtance in ſuch uncommon diſorders as thoſe of diſtorted limbs. Therefore, if application is made at all, it is made to itinerants and quacks; who, after having made what they can of the credulous parents, generally leave the unhappy victims in a worſe ſtate than that in which they found them.

This diſorder may be occaſioned by different cauſes; by a morbid ſtate of the bones, or contraction of the muſcles; by original mal-conformation, or wrong poſition in the womb. But from whatever cauſe the diſtortion may proceed, the reducing them to the natural poſition muſt be accompliſhed by nearly the ſame means; that is, by judicious preſſure upon the convex ſide. In ſome caſes the diſorder lies in the ankle joint, while the leg and knee are perfectly natural; in others, and perhaps, the greateſt number, it is occaſioned by a bending of the bones of the leg, by which the toes are turned either out or in, according as the bones are bent to the one ſide or to the other.

A caſe of the firſt kind, where the diſorder lay principally in the ankle joint, occurred to me lately. J. Wardrop, a child about ſeven weeks old, had her feet ſo diſtorted, that the toes of the one pointed directly to the ankle of the other. Upon the external ankle of the left, and down the back of the foot, there was a conſiderable riſing, with a correſponding hollow in the other ſide, which ſhowed that the bones of the ankle joint were ſhifted completely out of their place. The right foot had more of the natural ſhape, but, with regard to the directions of the toes, it was as much diſtorted as the other. The ſoles of both were conſiderably turned, ſo that, when walking, ſhe would have gone directly upon the outſides of her feet.

From

From her birth till application was made to me, various attempts had been made to correct the deformity; ſhoes, boots, and bandages of all kinds had been tried, but without ſucceſs. Some of them it was found impoſſible to retain for any length of time, or if retained, they hurt the feet ſo much, that they were under the neceſſity of removing; others, and that the greateſt number, if they did no harm, they did as little good. When ſhe was firſt put under my care, as I had never ſeen a caſe of the ſame kind treated before, I began to turn over every volume upon ſurgery I could lay my hands on; but finding nothing to my purpoſe ſave in general terms, I began to think for myſelf, when I fell upon the following contrivance, which I am happy to ſay, has anſwered the purpoſe extremely well.

Figure I. repreſents the machine containing the limbs of the child. AAAA, the legs and feet of the machine, which ought to be made of white iron, hardened leather, or ſome other firm ſubſtance, ſtrong enough to retain the limbs in a proper poſition. BB, two pieces of ſoft leather fixed to the inſide of the legs of the machine, laced upon the foreſide of the limbs of the child to retain them in their place: the toes ought to be left uncovered, by this means we can judge more accurately of the poſition of the feet. CC, two pieces of iron with joints in the middle; theſe connect the limbs together, and by the joints allow the toes to be ſeparated from one another, or brought nearer, according as the caſe may require. DD, two curved pieces of iron fixed to the toes of the machine, the one having a long ſlit, the other a ſcrew, by which means the toes can be fixed in any poſition we pleaſe.

To prevent the child's feet from being hurt by preſſure againſt the inſide of the machine, let them be rolled with a ſoft flannel roller from the toes up to the knee, and frequently anointed with ſome emolient liniment. At firſt, the machine ſhould be applied only for a few hours at once; but after ſome time, it may be kept on for two or three days without doing the leaſt injury to the child's feet, and with conſiderably more advantage than when it is frequently removed. In the above caſe, at one time the machine was kept on without being changed for upwards of four days; and I obſerved, that in this interval they made more progreſs than ever they had done before in the ſame time, when the machine was changed daily. To keep the child quiet while we were putting on the inſtrument, and through the night, I found it neceſſary to uſe a gentle opiate once in the twenty-four hours. This, however, did no hurt to the child's health; and as ſoon as the operation was finiſhed, we gave it up entirely.

In turning out or in the toes it ſhould be done by degrees, and not all at once: this may be eaſily managed by means of the ſcrew H. By making a ſmall advance every day, a ſhort time will bring them to the place we wiſh. And here it may be proper to obſerve, that whether the feet are turned out or in, it will be neceſſary to bring them about with the machine a little farther than we wiſh them to ſtand, for after it is finally removed they naturally recede a little.

I need ſcarcely obſerve too, that though the one foot only ſhould be diſtorted, it will be neceſſary to uſe a double machine, that is, a machine with two legs, one for the ſtraight limb as well as one for the crooked: for it is impoſſible to retain the diſtorted one in a proper poſition without connecting it to the other. In this way the cure will be done with the greateſt eaſe, and no injury whatever will be done to the ſound limb. In the caſe above related, although the machine was kept on for nearly two months, yet there was not a ſingle ſcratch or bruiſe upon her whole limbs, and all the while ſhe grew, and was as healthy as ever ſhe was, either before or ſince.

When I firſt thought of this inſtrument, I had no idea of its being applied to any other caſe but the *vari* and *valgi*, and theſe only when the diſorder happened to lie in the ankle joint. But I have ſince thought, that by a ſmall alteration, it may be applied to all the different kinds of diſtorted limbs with conſiderable eaſe and advantage.

Figure II. repreſents the half of the machine, in every reſpect ſimilar to the one above deſcribed, ſave only, that one of its ſides from the heel up is removed, and in place of it a ſpring, the under end of which is fixed at E, to the ſide of the heel; the other, by means of the ſtrap F, to the top of the machine. The convex ſide of the ſpring, ſtuffed and covered with ſoft leather, being applied to the convex ſide of the limb, by means of the ſtrap F. any degree of preſſure can be made which the practitioner may judge neceſſary. The ſide of the leg of the machine oppoſite the ſpring, muſt have a head as repreſented at G, to reſt againſt the outer or inner condyle of the femur, according as the caſe may require. If the bones are bent forward, as is often the caſe, by fixing the ſpring upon the foreſide, and cauſing it to preſs upon the convexity of the limb, they may be cured in the ſame manner as above directed. By uſing a ſpring inſtead of ſtraps round the limb, as directed by Mr. Benjamin Bell, a more uniform and effectual preſſure can be made. With regard to the ſtrength of the ſpring, no general rule can be given; this muſt depend entirely upon the age of the patient, or the degree of force required to ſtraighten the limb.

Figure

Figure III. reprefents one leg of the machine without the child's limb, having a fpring fixed to the one fide of the heel, as above directed. At LL and II, there muft be two rows of fmall holes, to which the leather which furrounds the child's limbs muft be fixed. K reprefents a fcrew with which the fpring may be fixed inftead of the ftrap F. I am,

GENTLEMEN,

Your humble fervant,

*Paifley, April* 3, 1800. R. WATT.

P. S. At the time when the above was written and ready to be fent off, J. Wilfon, a child about eight days old, having her feet diftorted in the fame manner as above defcribed, but confiderably worfe, was put under my care; I treated her in the fame manner and with equal fuccefs. In the courfe of two months fhe was completely cured, and her feet are now as natural and well-fhaped as any other child's of the fame age. At the firft, the offa calcium feemed either to be extremely fmall, or completely wanting; but after applying the machine for a few weeks, they grew and became perfectly well formed. The protuberances too upon the outfides of her feet, produced by the difplacing of the metatarfal bones, difappeared, and the hollow below the internal ankle filled up. From the fuccefs in treating thefe two cafes, I am inclined to think that there is no kind of diftorted limb, however formidable it may appear, if taken in time, but may be cured by the fame means.

## Figures and Tables

**Fig. 1. Fig. 2. Fig. 3. f1:**